# Effect of prior cancer on survival outcomes for patients with advanced prostate cancer

**DOI:** 10.1186/s12894-021-00792-w

**Published:** 2021-02-17

**Authors:** Yechen Wu, Xi Chen, Duocheng Qian, Wei Wang, Yiping Zhang, Jinxin Hu, Jun Zhu, Qiang Wu, Tinghu Cao

**Affiliations:** 1 Department of Urology, Baoshan Branch, Shuguang Hospital Affiliated to Shanghai University of Traditional Chinese Medicine, Shanghai, 201900 People’s Republic of China; 2 Department of Urology, Tongji Hospital, Tongji University School of Medicine, Shanghai, 200065 People’s Republic of China; 3 Department of Urology, Shanghai Forth People’s Hospital Affiliated to Tongji University School of Medicine, Shanghai, 200434 People’s Republic of China; 4 Department of Urology, Tongji Hospital, Tongji University School of Medicine, Shanghai, 200065 People’s Republic of China

**Keywords:** Advanced prostate cancer, Prior cancer, Survival, SEER, Trial eligibility

## Abstract

**Background:**

A history of prior cancer commonly results in exclusion from cancer clinical trials. However, whether a prior cancer history has an adversely impact on clinical outcomes for patients with advanced prostate cancer (APC) remains largely unknown. We therefore aimed to investigate the impact of prior cancer history on these patients.

**Methods:**

We identified patients with advanced prostate cancer diagnosed from 2004 to 2010 in the Surveillance, Epidemiology, and End Results (SEER) database. Propensity score matching (PSM) was used to balance baseline characteristics. Kaplan–Meier method and the Cox proportional hazard model were utilized for survival analysis.

**Results:**

A total of 19,772 eligible APC patients were included, of whom 887 (4.5 %) had a history of prior cancer. Urinary bladder (19 %), colon and cecum (16 %), melanoma of the skin (9 %) malignancies, and non-hodgkin lymphoma (9 %) were the most common types of prior cancer. Patients with a history of prior cancer had slightly inferior overall survival (OS) (AHR = 1.13; 95 % CI [1.02–1.26]; P = 0.017) as compared with that of patients without a prior cancer diagnosis. Subgroup analysis further indicated that a history of prior cancer didn’t adversely impact patients’ clinical outcomes, except in patients with a prior cancer diagnosed within 2 years, at advanced stage, or originating from specific sites, including bladder, colon and cecum, or lung and bronchus, or prior chronic lymphocytic leukemia.

**Conclusions:**

A large proportion of APC patients with a prior cancer history had non-inferior survival to that of patients without a prior cancer diagnosis. These patients may be candidates for relevant cancer trials.

**Supplementary Information:**

The online version contains supplementary material available at 10.1186/s12894-021-00792-w.

## Background

Prostate cancer represents the most common malignancy in men, accounting for estimated 164,690 new cases in the United States, in 2018 [1]. According to the latest statistical report, prostate cancer still represents the second most common cause of death in men (9 % of all cancer deaths) [2]. Although great advances have been made in the past several years, huge challenges still exist in patients with advanced prostate cancer, which is still associated with substantial morbidity and mortality, particularly in patients who develop resistance after multiple lines of therapy. The National Comprehensive Cancer Network hold the opinion that the best management for those patients with advanced disease is clinical trials, because well-designed clinical trials are pivotal for exploring new treatments and improving patients’ clinical outcomes. Unfortunately, patients who had a prior cancer history are often excluded by strict eligibility criteria in cancer trial. Given the dramatical increase in the number of cancer survivors as well as the decreasing cancer mortality rate, the exclusion criterion may limit the accrual and generalizability of clinical trials, and thus leaves many pivotal clinical issues unanswered [3, 4].

It was reported that up to 18 % of lung cancer patients were unconditionally excluded by over 80 % of lung cancer trials due to a history of prior cancer [5]. This practice is mainly due to concerns regarding to prior treatment interference and its survival impact, though little evidence clearly support this assumption. However, a previous retrospective study made by Laccetti et al. reported that a prior cancer history did not adversely affect survival of patients with advanced lung cancer, regardless of different stage or types of prior cancer [6]. Another study also suggested that the prognosis of patients with uterine papillary serous carcinoma was not affected by a prior breast cancer and tamoxifen exposure [7]. On the contrary, it was also reported that the overall survival were significantly lower in breast cancer patients as the second primary cancer than in that of patients with breast cancer as the primary cancer [8]. These different results implied that the survival impact of a prior cancer may vary among different cancer types. However, until recently, it remain unknown whether a history of prior cancer affects the clinical outcomes of APC patients.

Therefore, we conducted this study to assess the prevalence, types, timing, and prognostic impact of a prior cancer diagnosis on patients who developed advanced prostate cancer as a second primary malignancy by using the SEER database. Our finding may provide implications for exclusion criteria of relevant clinical trial.

## Methods

### Data source and case selection

The SEER*Stat software (v. 8.3.6.1) was utilized to extract data from the custom SEER database [Incidence- SEER 18 Regs Custom Data (with additional treatment fields), Nov 2018 Sub (1975–2016 varying)], which covers approximately 28 % of the United States population [9]. We included patients who were diagnosed with advanced prostate cancer (site code C) from 2004 to 2010 in order to ensure a 5-year follow-up at least. Patients were eligible if they had stage IV prostate cancer (N1M0 or M1) according to the 8th edition of the AJCC Cancer Staging Manual. Only patients with a single primary tumor or patient who had exactly one prior tumor were included. Other exclusion criteria were listed as follows: (1) patients whose prior cancer was prostate cancer; (2) patients with incomplete follow-up; (3) patients with only death certificates or autopsy records; (4) patients whose diagnosis time of malignancy was not known.

### Covariates

Multiple variables including demographic characteristics (diagnosed year, age, race, and marital status), disease characteristics (Seer stage, histologic grade, and prior cancer type), and treatment modalities (surgery, chemotherapy and radiotherapy). Marital status was categorized as single, married and other status (divorced, widowed, separated and domestic partner). The record of SEER sequence number was used to determine the prior cancer diagnosis. For example, patients who had only one primary tumor were recorded as “00”. For patients with multiple malignancies, the sequence number of “01” represented the first tumor, and “02” represented the second one, and so forth. We then calculated the timing, namely the time interval between two cancer record, by subtracting the diagnosis date of the prior cancer from that of index prostate cancer. Detections of vital status and cause-specific death classification were used to define the primary outcomes including overall survival (OS) and cancer-specific survival (CSS).

### Statistical analysis

Pearson chi-square test was utilized to compare clinicopathologic characteristics between patients with or without prior cancer. The propensity score matching (PSM) method was used to reduce the bias in baseline characteristics. Propensity scores were calculated based on variables including age, diagnosed year, race, marital status, histological grade, surgery, chemotherapy, and radiotherapy, with a ratio of 1:1 and a calliper of 0.2 [10]. Kaplan–Meier method and log-rank test were utilized to compare differences of OS in patients with no prior cancer vs. any prior cancer, before and after PSM. Multivariate Cox proportional hazards models were also built to determine whether prior cancer affects patients’ prognosis independently. Descriptive statistic, Pearson Chi-square test, and Cox proportional hazards model were performed using SPSS 24.0 (IBM Corp). The Kaplan–Meier plot and log-rank test were plotted or conducted by using R software version 4.0.0. A 2-sided P value of < 0.05 was considered as statistical significance unless otherwise stated.

## Results

**Fig. 1:**
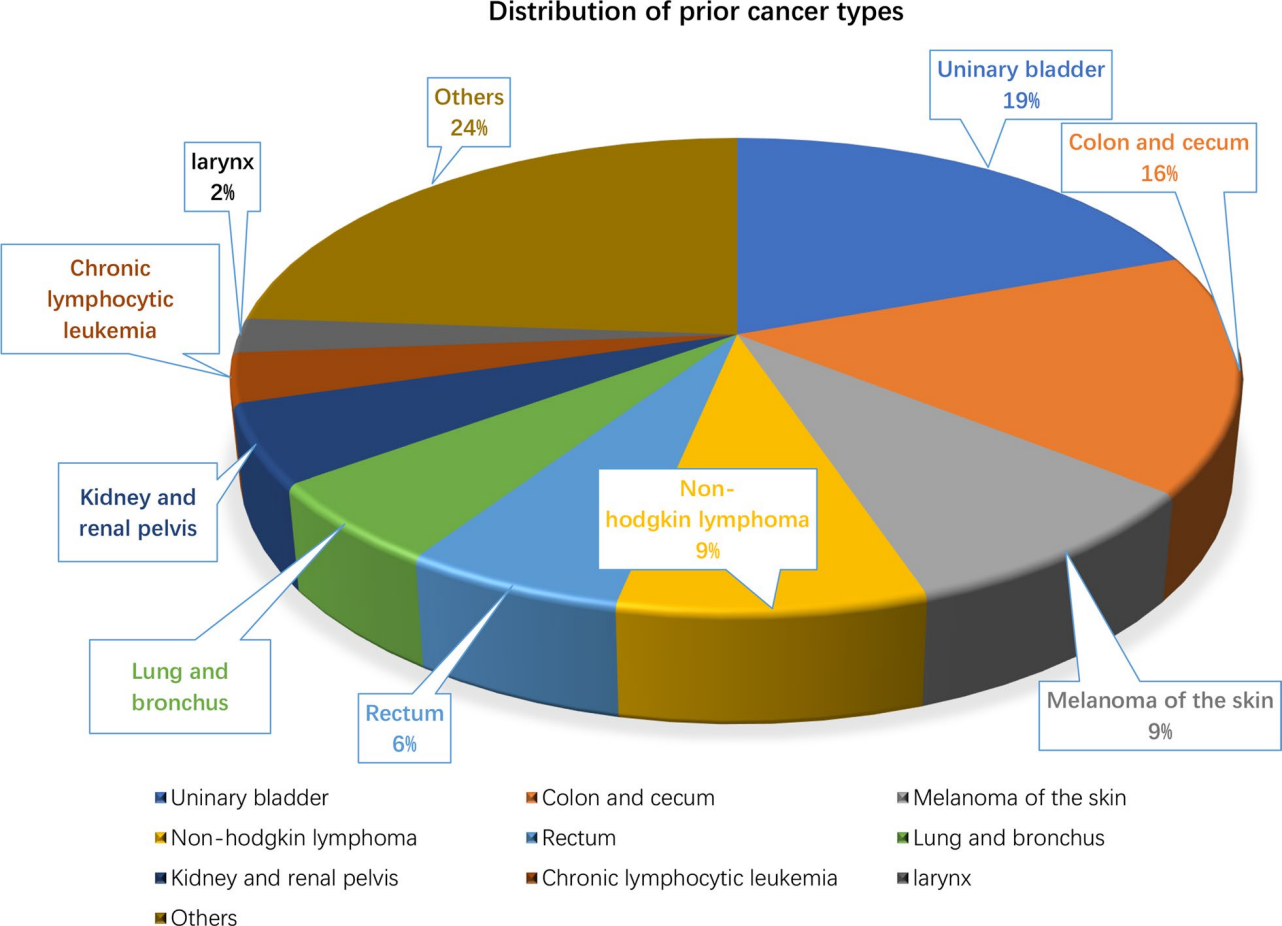
Distributions of prior cancer types in patients with advanced prostate cancer

A total of 19,772 eligible APC patients were extracted from SEER database, of whom 887 (4.5 %) carried a history of prior cancer. As shown in Table 1, the median age at prior cancer diagnoses was 70 years old, and that of subsequent APC was 77 years old. The median (interquartile range, IQR) time interval between two cancer diagnoses was 49 (22-95.5) months (Table 1). The Table 2 indicated that a history of prior cancer was more common among the elderly (75.6 years vs. 69.3 year), black (84.7 % vs. 75.4 %), and married (65.8 % vs. 59.8 %) individuals. After propensity score matching (PSM), all the baseline characteristics between patients with or without prior cancer history were balanced (Table 2). Figure 1 showed that the most common types of prior cancer in APC survivors included urinary bladder (19 %), colon and cecum (16 %), melanoma of the skin (9 %), and non-hodgkin lymphoma (9 %).

**Table 1:** Summary description of demographic and clinical factors

At prior cancer diagnosis		At advanced prostate cancer diagnosis	
Variable	Value	Variable	Value
Age, years		Age, years	
Mean	69.5	Mean	75.6
Median (IQR)	70 (27–78)	Median (IQR)	77 (68–84)
Marital status, n (%)		Marital status, n (%)	
Single	69 (7.8)	Single	73 (8.2)
Married	616 (69.4)	Married	584 (65.8)
Other status	147 (16.6)	Other status	182 (20.5)
Unknown	55 (6.2)	Unknown	48 (5.5)
Seer stage, n (%)		Seer stage, n (%)	
In situ	66 (7.4)	In situ	N/A
Localized	323 (36.4)	Localized	N/A
Regional	70 (7.9)	Regional	241 (27.2)
Distant	140 (15.8)	Distant	646 (72.8)
Unknown	288 (32.5)	Unknown	N/A
Interval between diagnoses, months		Follow up from diagnosis of gastric cancer, months	
Mean	74.4	Mean	37.2
Median (IQR)	49 (22–95.5)	Median (IQR)	23 (8.3–57.8)

*IQR* interquartile range

**Table 2:** Baseline characteristics of patients with advanced prostate cancer in the original/matched data sets (N = 19,772)

	Original data set			Matched data set		
Characteristics	No prior cancer N = 18,885 (%)	With prior cancer N = 887 (%)	P value	No prior cancer N = 887 (%)	With prior cancer N = 887 (%)	P value
Age (years), mean (SD)	69.3 (11.5)	75.6 (10.3)	< 0.001	75.8 (10.7)	75.6 (10.3)	0.661
Year of diagnose(%)			0.001			0.393
2004–2007	10,185 (53.9)	430 (48.5)		448 (50.5)	430 (48.5)	
2008–2010	8700 (46.1)	457 (51.5)		439 (49.5)	457 (51.5)	
Race			0.025			0.088
White	3442 (18.2)	85 (9.6)		101 (11.4)	85 (9.6)	
Black	14,231 (75.4)	751 (84.7)		717 (80.8)	751 (84.7)	
Others/Unknown	1212 (6.4)	51 (5.7)		69 (7.8)	51 (5.7)	
Marital status			< 0.001			0.174
Single	2636 (14.0)	73 (8.2)		94 (10.6)	73 (8.2)	
Married	11,292 (59.8)	584 (65.8)		543 (61.2)	584 (65.8)	
Others status ^a^	3742 (19.8)	182 (20.5)		198 (22.3)	182 (20.5)	
Unknown	1215 (6.4)	48 (5.4)		52 (5.9)	48 (5.4)	
Grade			< 0.001			0.608
Grade I	1209 (6.4)	79 (8.9)		70 (7.9)	79 (8.9)	
Grade II	13,411 (71.0)	555 (62.6)		573 (64.6)	555 (62.6)	
Grade III	4265 (22.6)	253 (28.5)		244 (27.5)	253 (28.5)	
Surgery			0.304			0.159
No/unknown	13,951 (73.9)	669 (75.4)		694 (78.2)	669 (75.4)	
Yes	4934 (26.1)	218 (24.6)		193 (21.8)	218 (24.6)	
Chemotherapy			0.468			0.413
No/unknown	17,843 (94.5)	833 (93.9)		835 (94.1)	833 (93.9)	
Yes	1042 (5.5)	54 (6.1)		52 (5.9)	54 (6.1)	
Radiotherapy			0.013			0.639
No/unknown	14,191 (75.1)	699 (78.8)		707 (79.7)	699 (78.8)	
Yes	4694 (24.9)	188 (21.2)		180 (20.3)	188 (21.2)	

^a^Other status including divorced, widowed, separated or domestic partner

**Fig. 2:**
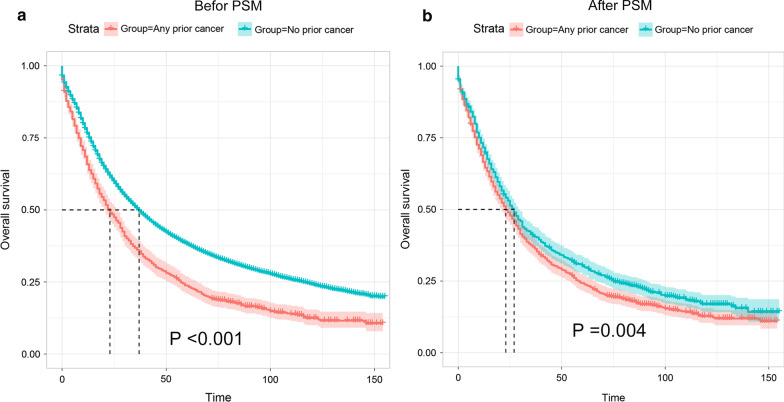
Kaplan–Meier survival curves of prior cancer impact on the overall survival (OS) in advanced prostate cancer patients with or without prior cancer. **a** The OS analysis before Propensity score matching (PSM); **b** The OS analysis after PSM

The Kaplan–Meier plot was utilized to compare the OS between patients who had, or had not prior cancer. As shown in Fig. 2a, the OS of patients with a history of prior cancer was dramatically lower (P < 0.001) than that of patients without a prior cancer history. After PSM, the Kaplan–Meier plot still showed a worse survival for patients who had a history of prior cancer, presenting a potential adverse effect of a prior cancer history on clinical outcome (P = 0.004) of patients with subsequent advanced prostate cancer (Fig. 2b).

**Fig. 3:**
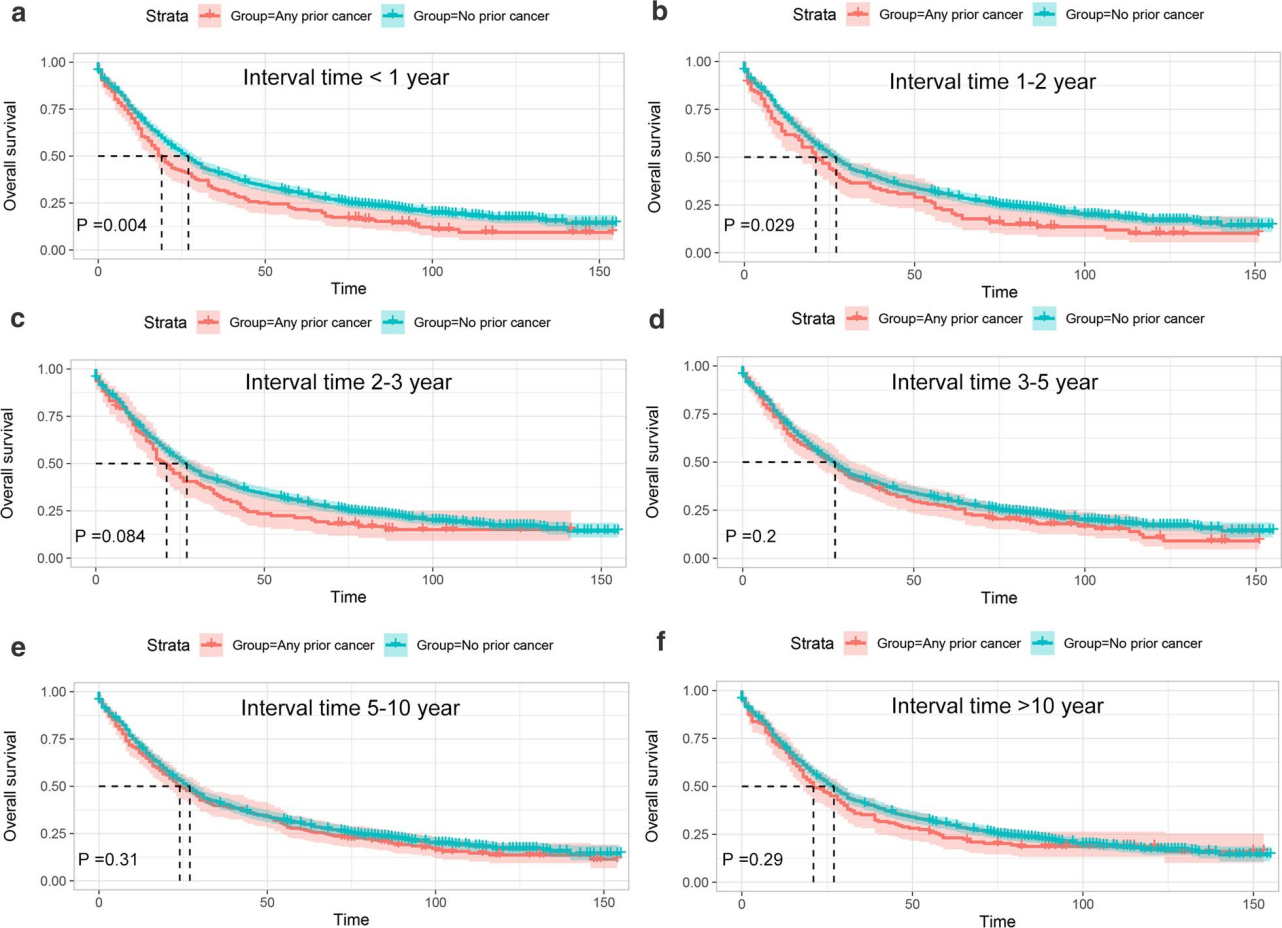
Kaplan–Meier survival curves of prior cancer impact on the overall survival (OS) stratified by timing of prior cancer in patients with advanced prostate cancer. **a** The OS analysis with time interval less than 1 year; **b** The OS analysis with time interval between 1–2 year; **c** The OS analysis with time interval between 2–3 year; **d** The OS analysis with time interval between 3–5 year; **e** The OS analysis with time interval between 5–10 year; **f** The OS analysis with time interval longer than 10 years

**Fig. 4:**
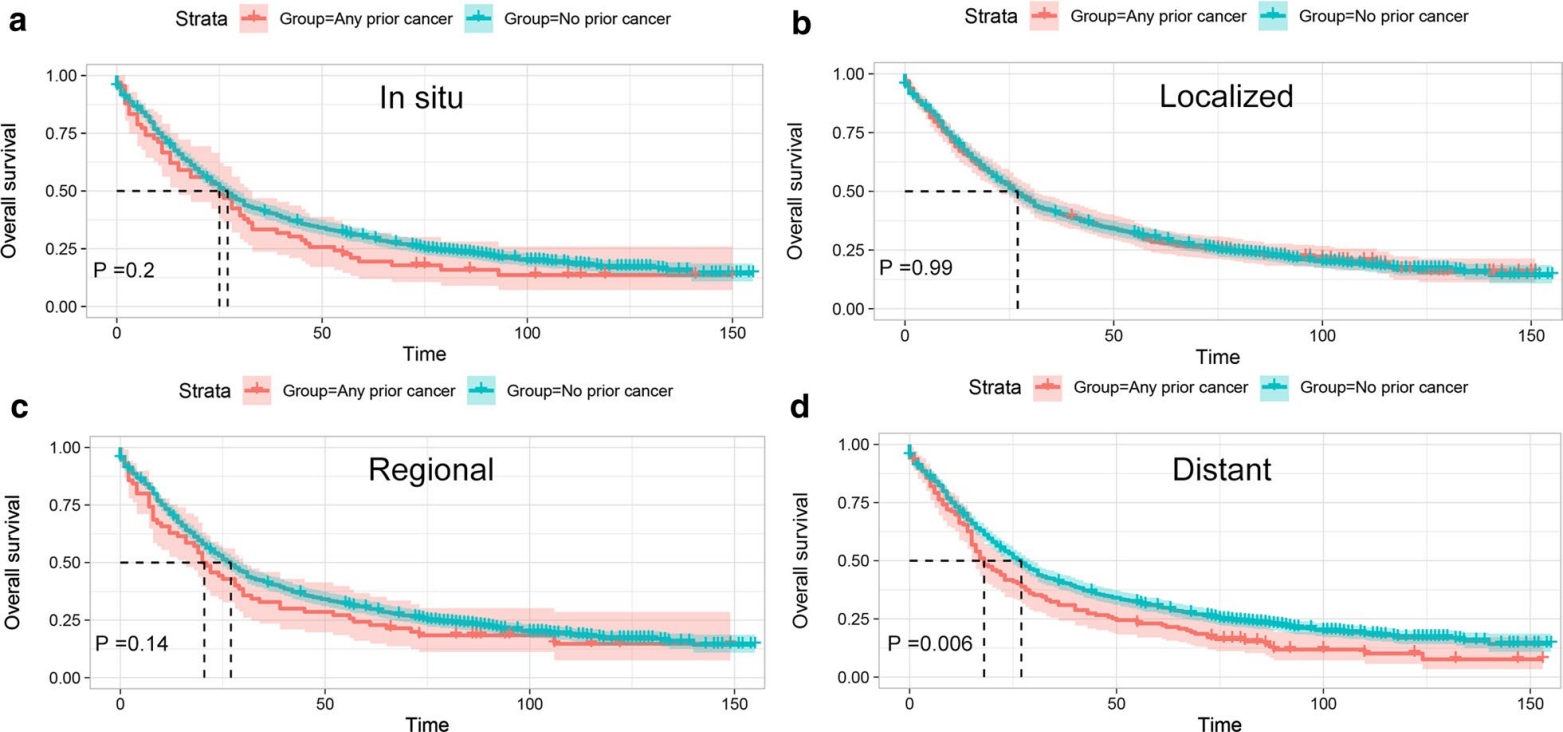
Kaplan–Meier survival curves of prior cancer impact on the overall survival (OS) stratified by stage of prior cancer in patients with advanced prostate cancer. **a** The OS analysis with prior cancer at *in situ* stage; **b** The OS analysis with prior cancer at localized stage; **c** The OS analysis with prior cancer at regional stage; (D) The OS analysis with prior cancer diagnosed at advanced stage

**Fig. 5:**
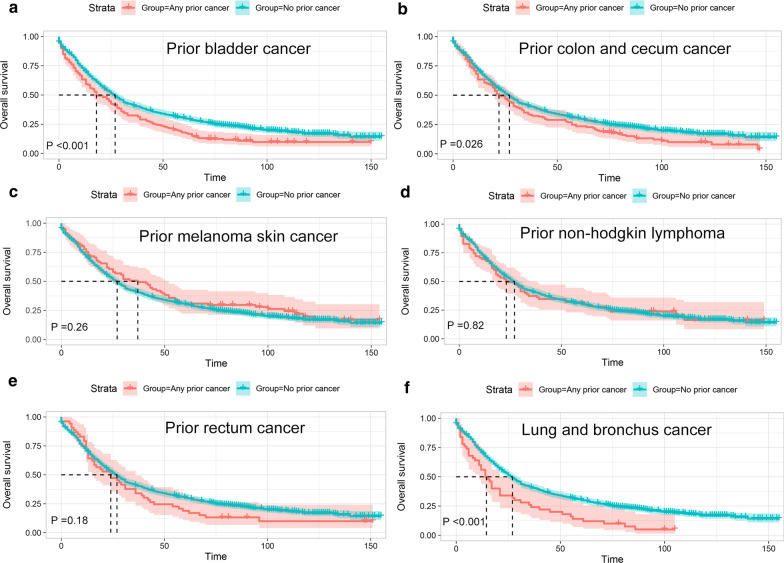
Kaplan–Meier survival curves of prior cancer impact on the overall survival (OS) stratified by different types of prior cancer in patients with advanced prostate cancer. **a** The impact of prior bladder cancer on OS; **b** The impact of prior colon and cecum cancer on OS; **c** The impact of prior melanoma skin cancer on OS; **d** The impact of prior non-hodgkin lymphoma on OS; **e** The impact of prior rectum cancer on OS; **f** The impact of prior lung and bronchus cancer on OS

In order to further investigate the survival impact of prior cancer, subgroup analyses were subsequently performed for APC patients stratified by timing (time interval), stage categorization and types of prior cancer. As shown in Fig. 3, we found that patients who had a prior cancer diagnosis with time interval of 2 years or longer showed non-inferior prognosis (P > 0.05) to that of patients without a prior cancer diagnosis. We also found that only prior cancer with advanced stage had significantly adverse impact on OS, while no survival detriment was observed in patients with a prior cancer diagnosed at in situ, localized, or regional stage (Fig. 4). Furthermore, our results also showed that a prior bladder, colon and cecum, lung and bronchus cancer, or CLL had a dramatically (P < 0.05) adverse effect on survival of patients with subsequent advanced prostate cancer (Fig. 5 and Additional file 1: Fig. S1). However, patients whose prior cancers originating from other sites presented similar OS as compared with that of patients without a prior cancer diagnosis.

After adjusted for age, race, marital status, histologic grade, and treatment modalities, the multivariate Cox regression analysis showed that a prior cancer history was significantly associated with worse OS (HR = 1.13; 95 % CI [1.02–1.26]) for patients with advanced prostate cancer (Table 3). Similar to the Kaplan–Meier method, the multivariate Cox analysis for the subgroup analysis further demonstrated that only prior cancer with time interval less than 1 years (HR = 1.35; 95 % CI [1.11–1.66]) or within 1–2 years (HR = 1.27; 95 % CI [1.02–1.57]), with prior cancer diagnosed at advanced stage (HR = 1.31; 95 % CI [1.08–1.58]), or with prior cancer of bladder (HR = 1.39; 95 % CI [1.16–1.66]), colon and cecum (HR = 1.23; 95 % CI [1.02–1.48]), lung and bronchus cancer (HR = 1.54; 95 % CI [1.15–2.08]) or CLL (HR = 1.47; 95 % CI [1.01–2.15]) significantly affected the prognosis of patients with advanced prostate cancer. Nevertheless, patients with other different timing, with other stage categorization or with other types of prior cancer had non-inferior survival to that of patients without a prior cancer diagnosis. Our result also showed that patients with a history of prior cancer presented non-inferior or even slightly superior prostate cancer-specific survival to patients without a prior cancer history. Detailed data can be seen in Table 3.

**Table 3:** Multivariable Cox analysis for advanced prostate patients with prior cancer (vs. no prior cancer)

Characteristic	Overall survival		Prostate cancer-specific survival	
	HR (95 % CI)	P	HR (95 % CI)	P
All patients	1.13 (1.02–1.26)	0.017	0.93 (0.81–1.06)	0.248
Part I: Prior cancer timing (vs. no prior cancer)				
≤ 1 year	1.35 (1.11–1.66)	0.003	0.80 (0.60–1.08)	0.152
1–2 year	1.27 (1.02–1.57)	0.033	0.97 (0.72–1.29)	0.818
2–3 year	1.18 (0.93–1.49)	0.176	0.78 (0.56–1.09)	0.150
3–5 year	1.14 (0.94–1.37)	0.180	0.97 (0.77–1.23)	0.792
5–10 year	1.05 (0.89–1.24)	0.535	0.93 (0.76–1.15)	0.512
>10 year	1.11 (0.92–1.33)	0.270	1.09 (0.88–1.35)	0.432
Part II: Prior cancer stage (vs. no prior cancer)				
In situ	1.13 (0.87–1.47)	0.365	0.90 (0.64–1.27)	0.539
Localized	1.05 (0.91–1.21)	0.537	0.87 (0.73–1.05)	0.139
Regional	1.27 (0.97–1.68)	0.083	0.91 (0.70–1.19)	0.488
Distant	1.31 (1.08–1.58)	0.007	0.77 (0.51–1.16)	0.206
Part III: Prior cancer type (vs. no prior cancer)				
Urinary bladder	1.39 (1.16–1.66)	< 0.001	0.98 (0.77–1.26)	0.880
Colon and cecum	1.23 (1.02–1.48)	0.034	0.67 (0.67–1.11)	0.243
Melanoma of the skin	1.02 (0.70–1.19)	0.502	0.93 (0.68–1.26)	0.618
Non-hodgkin lymphoma	1.16 (0.89–1.52)	0.277	1.22 (0.89–1.65)	0.213
Rectum	1.10 (0.81–1.48)	0.546	0.78 (0.52–1.19)	0.250
Lung and bronchus	1.54 (1.15–2.08)	0.004	0.90 (0.58–1.42)	0.658
Kidney and renal pelvis	0.79 (0.57–1.09)	0.154	0.70 (0.47–1.06)	0.096
Chronic lymphocytic leukemia	1.47 (1.01–2.15)	0.046	1.10 (0.66–1.85)	0.707
larynx	1.06 (0.64–1.76)	0.819	0.94 (0.50–1.78)	0.850
Others	1.05 (0.89–1.24)	0.576	0.92 (0.77–1.14)	0.447

*HR* hazard ratio, *CI* confidence interval

The multivariate analysis was adjusted for age, race, marital status, histologic grade, and treatment modalities (surgery, chemotherapy, and radiation)

## Discussion

This study focused on survival impact of a prior cancer history on APC patients. Approximately 4.5 % of patients with advanced prostate cancer had a prior cancer history. Those patients showed a worse prognosis in comparison with patients without a prior cancer diagnosis. Nevertheless, subgroup analyses indicated that a history of prior cancer didn’t adversely affect patients’ survival, except for patients with prior cancer diagnosed within 2 year, or those with prior cancer diagnosed at advanced stage, or those with specific types of prior cancer, including bladder, colon and cecum, lung and bronchus cancer, or CLL .

Over the last few decades, the population of cancer survivors has been steadily increasing in the United States because of the aging of the population and the great advances in early detection and cancer treatment [11–13]. This population had a high risk of developing second primary cancer [14, 15]. Previous study reported that about one-tenth of younger adults and one-fourth of the elderly cancer patients had a prior cancer history [16].

Similar to other types of cancer, prostate cancer is frequently diagnosed as a second malignancy. In our study, we focused on assessing the survival impact of a prior cancer history on APC patients who are often candidates for clinical trials. We found that approximately 4.5 % of patients had exactly one non-prostate prior cancer before the diagnosis of APC. This proportion was similar to advanced breast cancer but lower than advanced lung cancer [6, 17]. Besides, similar to studies reported by Bluethmann et al. [16] and Murphy et al. [18], our study suggested that the elderly patients were more likely to have a history of prior cancer than the younger patients. The median time interval between advanced prostate cancer and prior cancers was approximately four years, which was longer than some cancers [3, 8]. The urinary bladder, colon and cecum, melanoma of the skin, and Non-hodgkin lymphoma exhibited as the most common types of prior cancer in the APC patients, of which the distribution differs from other cancers, such as nasopharyngeal cancer [3], lung cancer [19] and breast cancer [8].

The cancer trials exerts as a promising way for improving survivorship of advanced cancer patients. However, fewer than 5 % of patients can be enrolled in cancer trials due to the overly restrictive exclusion criteria [20], and a history of prior cancer was the commonly used one in most trials. This could mainly due to the widely accepted belief that a prior cancer can adversely affect patients’ survival, though no authoritative data have proved it. The stringent criteria may weed out a large number of patients who had urgent need, which could limit generalizability and lead to premature trial termination [4, 21]. Therefore, liberalizing the exclusion criteria, especially for a history of prior cancer, has been proposed by several working groups [5, 22]. Furthermore, the Food and Drug Administration (FDA) has introduced a draft guidance implying that patients who had a prior cancer history could generally be enrolled in clinical trials [23].

Our data indicated that the overall survival of APC patients with a history of prior cancer was significantly poorer than that of patients without a prior cancer history before and after PSM method. This result was consistent to the pan-cancer study by Zhou et al. [24]. However, subsequent subgroup analyses revealed that a prior cancer history could impair the survival of patients only when the interval time was less than two year. This time-frame finding was different from the study by Lin et al. that demonstrated no survival detriment in patients with advanced breast cancer who had prior cancer outside the timeframe of 4 years [17].

Our study also demonstrated that a prior cancer diagnosed at in situ, localized, or regional stage didn’t adversely affect the OS of APC patients. Moreover, subgroup analysis further showed that an inferior OS was only observed in APC survivors who had prior cancer originating from bladder, colon and cecum, or lung and bronchus, or prior CLL. In addition, these aforementioned results were further confirmed by the multivariable Cox analysis after adjusting for various clinicopathological variables. Hence, our data implied that a large number of APC patients who had a history of prior cancer may be eligible candidates for relevant cancer trials.

There are also several limitations in our study. First, other information such as efficacy and toxicity of treatment on prior cancer could not be considered due to lack of relevant data. Second, selection bias is inherent because of the intrinsic weaknesses of retrospective study. Therefore, further study is warranted to confirm the generality of our findings.

## Conclusion

In conclusion, a large number of APC patients have a prior cancer history. Only prior cancer diagnosed within two year, at advanced stage, or some specific prior cancer adversely affect APC patients’ survival. Therefore, for APC cancer patients with prior cancer history, broader inclusion criterion should be adopted to increase the accrual rate for the relevant clinical cancer trials.

## Supplementary Information

**Additional file 1:** Fig. S1. Kaplan-Meier survival curves of prior cancer impact on the overall survival (OS) stratified by different types of prior cancer in patients with advanced prostate cancer. (A) The impact of prior kidney cancer on OS; (B) The impact of prior chronic lymphocytic leukemia on OS; (C) The impact of prior larynx cancer on OS; (D) The impact of prior others cancer on OS.

## Data Availability

The datasets generated and/or analysed during the current study are available in the Surveillance, Epidemiology, and End Results Program repository, https://seer.cancer.gov/data/.

## Abbreviations

APC: Advanced prostate cancer
SEER: Surveillance, Epidemiology, and End Results
OS: Overall survival
CSS: Cancer-specific survival
PSM: Propensity score matching
HR: Adjusted hazard ratio
IQR: Interquartile range
CLL: Chronic lymphocytic leukemia
