# Supplementary figures and images for: Effect of prior cancer on survival outcomes for patients with advanced prostate cancer
Source: BMC Urol. 2021 Feb 17;21:26. doi: 10.1186/s12894-021-00792-w (PMC7891168; doi:10.1186/s12894-021-00792-w)

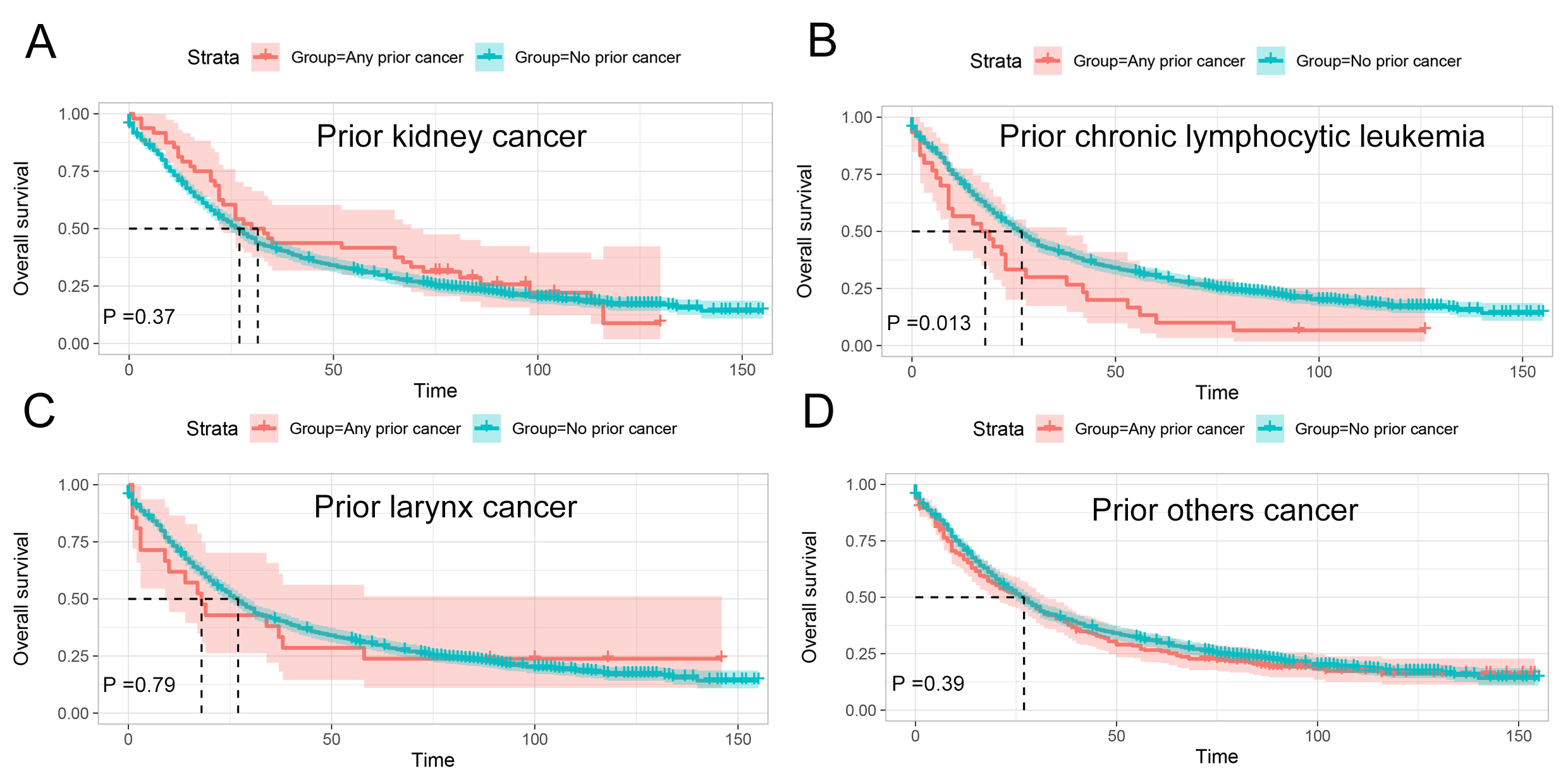

Supplement: Supplementary file 1 — Additional file 1: Fig. S1. Kaplan-Meier survival curves of prior cancer impact on the overall survival (OS) stratified by different types of prior cancer in patients with advanced prostate cancer. (A) The impact of prior kidney cancer on OS; (B) The impact of prior chronic lymphocytic leukemia on OS; (C) The impact of prior larynx cancer on OS; (D) The impact of prior others cancer on OS. [file 12894_2021_792_MOESM1_ESM.tif]
